# Study of spatial and temporal aging characteristic of catalyzed diesel particulate filter catalytic performance used for diesel vehicle

**DOI:** 10.1038/s41598-020-76634-w

**Published:** 2020-11-13

**Authors:** Yun-hua Zhang, Di-ming Lou, Pi-qiang Tan, Zhi-yuan Hu

**Affiliations:** School of Automotive Studies, Tongji University, Shanghai, 201804 China

**Keywords:** Pollution remediation, Chemical engineering, Porous materials

## Abstract

Catalyzed diesel particulate filters (CDPFs) have been widespread used as a technically and economically feasible mean for meeting increasingly stringent emissions limits. An important issue affecting the performance of a CDPF is its aging with using time. In this paper, the effects of noble metal loadings, regions and using mileage on the aging performance of a CDPF were investigated by methods of X-ray diffraction (XRD), X-ray photoelectron spectroscopy and catalytic activity evaluation. Results showed that aging of the CDPF shifted the XRD characteristic diffraction peaks towards larger angles and increased the crystallinity, showing a slowing downward trend with the increase of the noble metal loadings. In addition, the increase of the noble metal loading would slow down the decline of Pt and Pt^4+^ concentration caused by aging. The characteristic temperatures of CO, C_3_H_8_ conversion and NO_2_ production increased after aging, and the more the noble metal loadings, the higher the range of the increase. But noticeably, excessive amounts of noble metals would not present the corresponding anti-aging properties. Specifically, the degree of aging in the inlet region was the deepest, the following is the outlet region, and the middle region was the smallest, which were also reflected in the increase range of crystallinity, characteristic temperatures of CO, C_3_H_8_ conversion and NO_2_ production, as well as the decrease range of Pt and Pt^4+^ concentrations. The increase of aging mileage reduced the size of the aggregates of the soot and ash in CDPFs, however, improved the degree of tightness between particles. Meanwhile Carbon (C) concentration in the soot and ash increased with the aging mileage.

## Introduction

Diesel engines have been widely used in urban buses and heavy-duty trucks throughout the world for their strong power, good economy and high reliability^1,2^. Although the electrification of vehicle is coming, for a long period of time in the future, the market for diesel engines will be continuously growing in China due in large part to higher fuel efficiency compared to gasoline engines^3^. However, exhaust emissions including carbon monoxide (CO), hydrocarbons (properly indicated as CxHy, but typically expressed as HC), especially large amounts of particulate matter has brought about critical environmental and healthy issues^4^, resulting in the implementation of restrictions on diesel exhaust particulate matter (PM) and NOx emissions^5^. To meet the increasing emission regulations, a combination of diesel oxidation catalyst (DOC) and diesel particulate filter (DPF) is used as an efficient diesel engine after-treatment technology^6^. The upstream DOC can oxidize the CO and HC, the downstream DPF can trap the particle emission with a high efficiency up to 90%. The catalyzed diesel particulate filter (CDPF) manufactured by coating noble metal catalyst such as Pt and Pd et al. on the wall of DPF is being widely employed as an efficient diesel emission control technology^7^. It can not only oxidize CO, HC and increase the NO_2_ proportion by oxidizing NO^8^, but also filter the particles with a high efficiency^9^, and achieve a regeneration of the CDPF by oxidizing the trapped particles with a relatively low temperature^10^. The Pt and Pd have strong catalytic activity in oxidizing CO, HC and NO. More importantly, the Pt and Pd coated on the CDPF can substantially reduce the light-off temperature of the trapped soot from 450–550 °C to 250–300 °C with the presence of NO_2_, which enables the CDPF to conduct a passive regeneration at the exhaust temperature of diesel engine without extra temperature improvement measures^11^. In addition, adding Pd or Rh into the Pt-based catalyst can inhibit the sintering of Pt particles during aging and promote the re-dispersion of Pt particles, improving the anti-aging ability of the CDPF. Consequently, noble metal catalyst including Pt, Pd as well as Rh, plays a key role in the performance of the CDPF^12^. However, deterioration of catalyst used severely affects the performance of the CDPF^13^, leading to deactivate mechanisms including thermal aging^14^, chemical poisoning and mechanical damage^15^. With the continuous upgrading of fuel, the sulfur content is very low (≤ 10 ppm), causing less chemical poisoning of the catalyst^16^. Hence, thermal deactivation has always been the main reason for the deterioration of catalytic performance which is caused by the sintering and agglomeration of noble metal components as well as the sintering of washcoat surface area^17^. To investigate the deactivation of catalysts of the CDPF, different methods have been presented by scholars across the world. The cheapest and fastest method is thermal or hydrothermal oven ageing^18^, i.e. exposure to a constant high temperature for a defined time, but this method is not able to reflect the authentic aging process of the CDPF in real use^19^. On-engine durability test is also adopted to study the deterioration of catalytic performance, but there is no corresponding basis between the engine test time and the vehicle actually using mileage^20^. On-vehicle DPF durability evaluation is the most practicable even though this approach is typically time-consuming and, in many cases, cost prohibitive. Zhang et al. studied the deterioration performance of the CDPF based on an urban bus by monitoring the filter efficiency on particulate emissions with mileage, but further analysis about the changes in catalytic performance was not carried out in this study^21^. In fact, there are many factors affecting the deterioration of the CDPF catalytic activity, including noble metal loadings, the region in which catalyst is located and using mileage, et al. Liu et al. studied effects of coating on the performance of the CDPF and found that the increase in the amount of precious metal coated can improve its durability^22^. Tsuda et al. found that more Pt contained was efficient for enhancing the catalyst thermal durability^23^. Hauff et al. studied aging status of the catalyst with different Pt loadings and found that the catalytic activity is mainly related to the catalytically active surface which can be determined by Pt loadings^24^. Ren et al. found that the anti-aging characteristics of the catalyst increases with the precious metals loading coated, but excessive amounts of precious metals impair the anti-aging characteristics^25^. Except the precious metal loadings, Pt/Pd ratio in the catalyst of the CDPF also has an effect on the catalyst’s aging performance^26^. Besides, due to the effect of the exhaust flow and temperature, different regions in the CDPF presents different aging characteristics. Zhan et al. applied the on-engine tests to study durability of the DPF in different positions by monitoring the DPF thermal profiles of the inlet, mid-bed and rear-bed and found that the thermal shock is highest in the rear-bed of the DPF^27^. Bai et al. also found that the temperature peak appears at the rear-bed of the DPF, which will accelerate the aging of the coated catalyst^28^. The latest emission regulations put forward higher requirements for the durability of the CDPF^29^. The aging degree of the catalytic performance in the CDPF on-vehicle deepens with the using mileage, which can be reflected by the morphology and compositions of the soot and ash in the CDPF^30^. This can offer a new way for analyzing the effect of using mileage on the deterioration of CDPF. Although there have been many separate studies on the effects of noble metal loadings, regions or using mileage on the durability performance of CDPF, little studies were observed considering all the three influencing factors at the same time, especially using the method of on-vehicle aging. Therefore, this study aims to apply XRD, XPS and catalytic activity evaluation to analyze the effect of noble metal loadings on the durability performance of the CDPF using on-vehicle aging method. Meanwhile the aging characteristics of the CDPF in different regions were analyzed. Moreover, the microstructure and composition of the soot and ash in CDPFs with different aging mileage were investigated.

## Experimental method

### Specifications of CDPFs

In order to study the effect of noble metal loadings on the aging performance of CDPF, three different CDPFs were designed based on reference and engineering application experience^8,31^, and their specifications are shown in Table 1. The noble metal loadings of the CDPFs are 0.53, 1.71 and 1.24 g/L, and named No.1, No.2 and No. No.3 respectively. The CDPFs all adopted passive regeneration ways, and they were aged on the same series of 7.l L urban diesel buses.

**Table 1:** Specifications of CDPFs.

Item	Parameter		
	No.1	No.2	No.3
Substrate	Cordierite		
Thickness (mm)	0.35		
Cell density (cpsi)	200		
Porosity/%	55		
Average pore diameter (μm)	8–13		
Catalyst	Pt/Pd/Rh		
Pt:Pd:Rh	10:2:1		
Catalyst loadings (g/L)	0.53	0.71	1.24
Coatings	γ-Al_2_O_3_		

### Specifications of the vehicles

The designed CDPFs were installed on the same series buses, which were selected from a bus fleet on the same line. The main specifications of the buses were the same and they were presented in Table 2. All the buses ran on a fixed line with a daily mileage of about 200 km, and the transport load for every bus was almost the same during the mileage. Consequently, the effect of bus conditions, traffic and transport load on the passive regeneration degree of the CDPFs during the aging process were consistent.

**Table 2:** Main specifications of the buses.

Parameter	Value
Bus type	SUNWIN SWB6100V5
Curb weight (kg)	16,500
Engine type	Deutz D7E240
Capacity (L)	7.1
Maximum velocity (km/h)	85
Rated power (kW/ rpm)	177/2300
Maximum torque (N m/ rpm)	920/1700
Emission level	Euro III
Model year	2011

### Test methods and equipment

The XRD, XPS and catalytic activity evaluation were used to characterize the aging performance of the CDPFs. The XRD is a research method to obtain the information of material composition, internal atomic or molecular structure or morphology through the analysis of X-ray diffraction and diffraction patterns, which can accurately measure the crystal structure and crystal size^32^. The XPS is an effective method to measure the composition and chemical state of surface elements of the materials by quantitatively evaluating the binding energy peak intensity and peak displacement of different atoms or molecule^33^. The catalytic activity evaluation based on temperature-programmed reduction (TPR) system is an effective method to quantitatively analyze the catalytic activity of the catalyst^34^.

**Figure 1:**
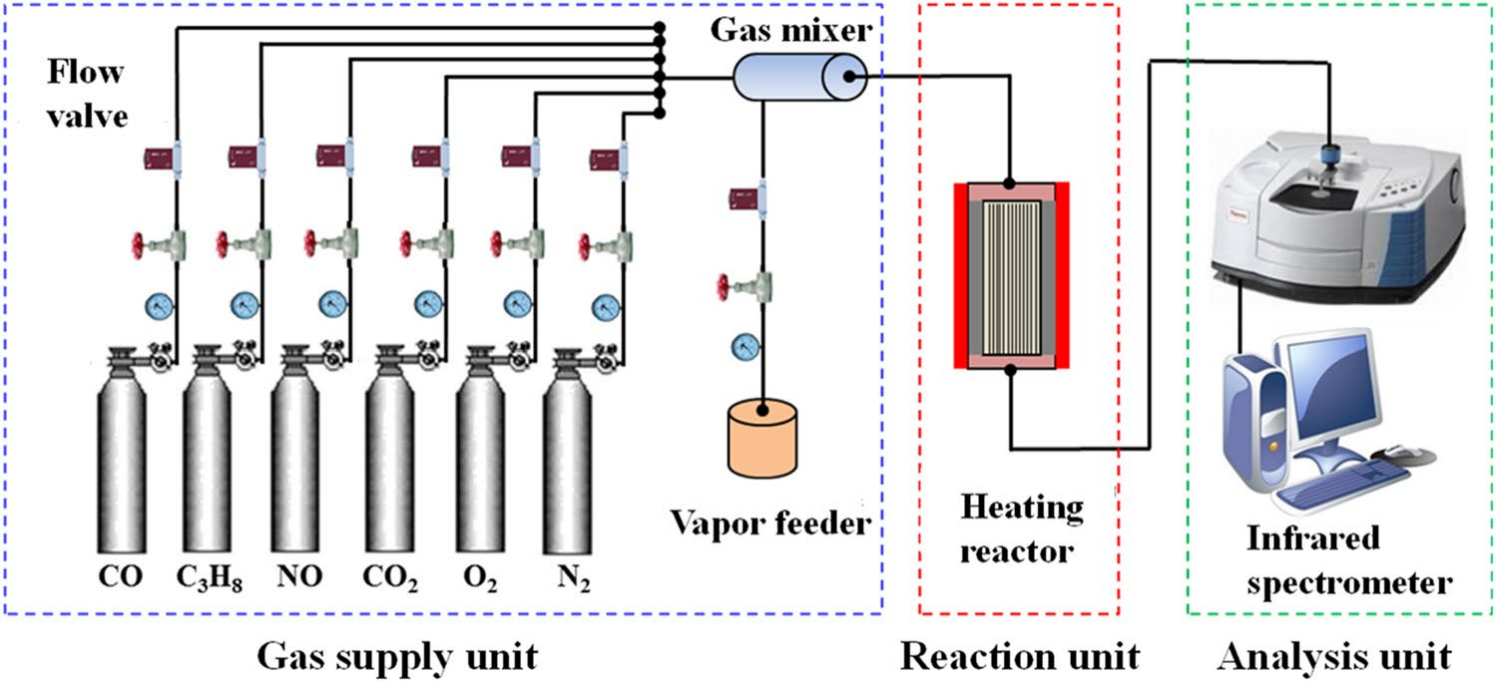
Catalytic activity evaluation system.

In this study, the XRD analysis was conducted to ascertain phase changes, crystallinity et al. of the CDPF samples by X ray diffractometer (D8 Advance, Bruker Axs Gmbh, Germany), the test accuracy is very high with angular reproducibility of ± 0.001°, and the test stability is ± 0.01%. The XPS analysis based on X ray photoelectron spectroscopy (PHI 5000C ESCA, PerkinElmer, USA) was carried out to evaluate the components, valence and distribution of noble metal elements in CDPF samples, the test resolution of this instrument is 0.1% and the angular reproducibility reaches ± 0.001°. The TPR system is used to evaluate the catalytic activity of the CDPF samples, this system consists of a gas supply unit, reaction unit and analysis unit as shown in Fig. 1. The gas supply unit provided the CO, C_3_H_8_ and NO components with concentration of 400, 400, 400 ppm, in addition, the O_2_ and N_2_ were provided as equilibrium gas. The saturated vapor was supplied from the vapor feeder, and the above gases were mixed through the gas mixer. Then the mixed gas entered the temperature-programmed heating reactor. The catalyst sample was input in the heating reaction and then analyzed by fourier transform infrared (FT-IR) spectrometer (Nicolet iS10, Thermofisher Scientific, USA), the resolution of this instrument reaches 0.0035 cm^−1^ and the test accuracy is ± 1%. In addition, the scanning electron microscope (SEM) and energy dispersive spectroscopy (EDS) instrument (Quanta 250FEG, FEI, USA) was used to conduct a morphology analysis of the soot and ash sampled from the CDPF, as well as a semi-quantitative analysis of the elements.

### Test program

When the CDPFs used for buses devices were prepared, the samples with the same catalyst parameters were also prepared. First, the fresh CDPF samples were cut into small samples with size of 10 mm × 10 mm × 50 mm, and the catalytic activity of the small samples were evaluated using TPR method. Meanwhile, the fresh CDPF samples were cut into small pieces to carry out the XRD and XPS tests. The standard CDPFs were installed to the same mode of buses. When the buses equipped with CDPFs run for about 30,000 km, the soot and ash in the No.2 CDPF were sampled by reverse purge method^35^, then the soot and ash were sampled for SEM test and energy dispersive spectrometer (EDS) test. When the buses run for 60,000 km, all the CDPFs were sampled with size of 10 mm × 10 mm × 50 mm in the central area by cutting method^36^, and the samples were tested using TPR to evaluate the catalytic activity. In addition, small pieces were also sampled for XRD and XPS tests. In addition, for No.2 CDPF, the soot and ash inside were also sampled by reverse purge method, and the soot and ash samples were used for SEM and EDS tests.

## Results and discussions

### The durability of the CDPFs with different noble metal loadings

In this part, the XRD, XPS and activity evaluation were carried out to analyze the durability of the CDPFs with different noble metal loadings.

#### XRD and crystallinity analysis

**Figure 2:**
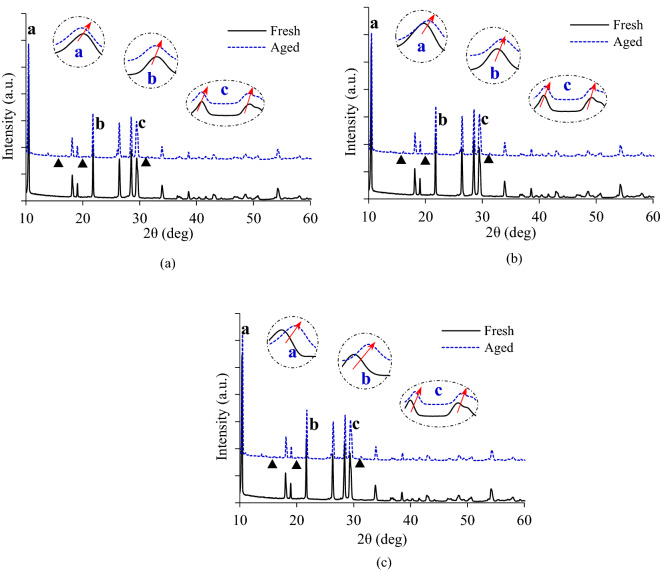
XRD results for fresh and aged CDPFs (**a**) No.1 CDPF, (**b**) No.2 CDPF, (**c**) No.3 CDPF.

Figure 2 shows the XRD diffraction patterns of CDPFs before and after 60,000 km’ aging. It can be seen that the XRD diffraction patterns of No.1, No.2 and No.3 CDPFs were similar. The characteristic diffraction peaks (marking a, b, c) all occurred in the ranges of 10°–11, 17°–22° and 25°–31° for the three CDPFs. After the CDPFs were used for 60,000 km, the peaks shifted towards large angles, which indicated that aging induced caused the lattice contraction of the sample and the reduction of the unit cell parameters. With the increase of the noble metal loadings, there was no obvious difference in the offset angle of XRD diffraction peaks. Besides, there were varying new peaks (marking filled triangle) for the aged CDPFs’ XRD diffraction patterns, which illustrated the formation of new crystalline phases. The possible reason was that the dispersed noble metals and metal oxides formed crystals with poor crystalline phase during aging, resulting in a decrease of the catalytic activity^37^.

**Figure 3:**
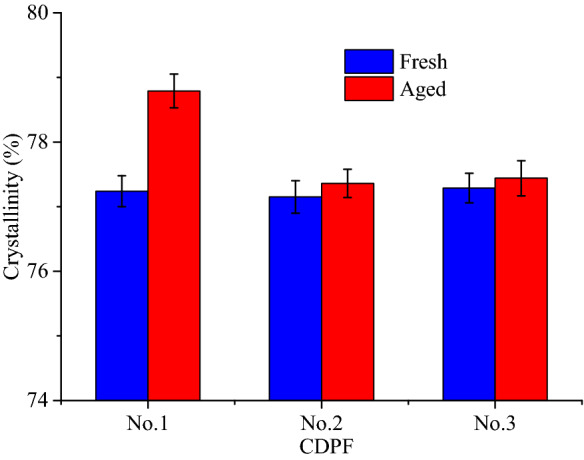
Crystallinity of the CDPFs before and after aging.

Figure 3 presents the crystallinity of the CDPFs from No.1 to No.3 before and after 60,000 km’ durability based on the Debye–Scherrer calculation method^38^. It can be seen that the crystallinity of the CDPFs increased after aging. Especially for No.1 CDPF, the crystallinity increased from 77.24% to 78.79%. While for No.2 and No.3 CDPFs, the increase of the crystallinity can be negligible. The increase of the crystallinity indicated that new crystalline phase formed. On the other hand, the growing of the grain of the substrate and the crystalline type tending to be complete after aging may also increase the crystallinity^39^.

#### Active element and valence states analysis

**Figure 4:**
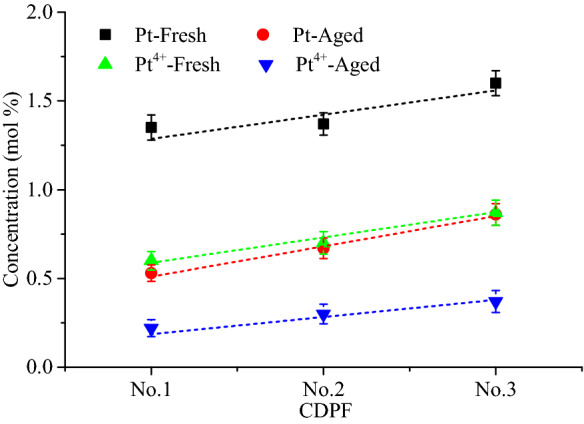
Pt and Pt^4+^ concentrations of fresh and aged CDPFs.

**Figure 5:**
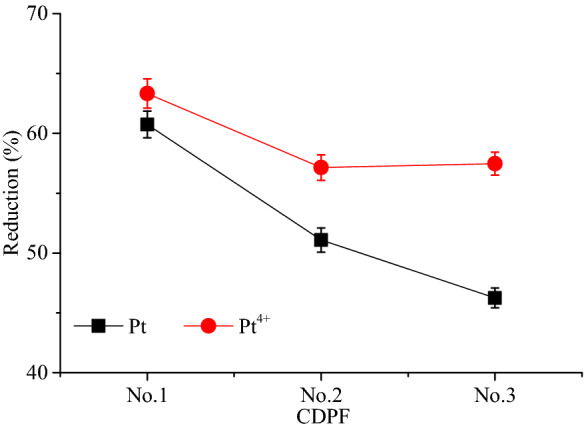
Reduction of Pt and Pt^4+^ concentrations of aged CDPFs.

The active elements Pt and Pt^4+^ concentrations shown in Fig. 4 were measured by XPS test. It can be seen that the Pt and Pt^4+^ concentrations increased linearly with the noble metal loadings. After aging, Pt and Pt^4+^ concentrations of the samples decreased significantly. Figure 5 presented the reduction of Pt and Pt^4+^ concentrations of the tress CDPFs after aging. It shown that the Pt and Pt^4+^ concentrations of No.1 CDPF decreased most of the three CDPFs, which reached 60.74% and 63.33%, respectively. For No.2 and No.3 CDPFs, the decreases of Pt and Pt^4+^ concentrations were nearly the same, but obviously, lower than that of No.1. Since the catalytic activity of the catalyst is directly related to the concentration of the noble metal, the results of active element and valence states analysis indicated that the higher the noble metal loadings, the slower the degradation of the CDPF catalytic performance.

#### Catalytic activity evaluation

In this part, the catalytic activities of CDPFs were studied by analyzing the temperatures corresponding to the conversion rates of CO, C_3_H_8_ and production rate of NO_2_ by CDPFs. The degradation rate of the catalytic properties of CDPFs is defined as:

1 $$ Deteriorating\;rate = \frac{T_{aged} - T_{fresh}}{{T_{aged}}} \times 100{\text{\% }} $$

where *T*_aged_ presents the temperatures of the CO, C_3_H_8_ conversion and NO_2_ production for aged CDPFs and *T*_fresh_ means the temperatures of the CO, C_3_H_8_ conversion and NO_2_ production for fresh CDPF.

**Figure 6:**
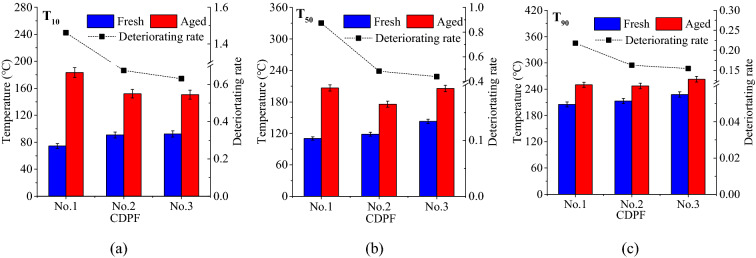
Characteristic temperatures and deteriorating rates of 10%, 50% and 90% CO conversion rates (**a**) 10% CO conversion rate − T_10_, (**b**) 50% CO conversion rate − T_50_, (**c**) 90% CO conversion rate − T_90_.

Figure 6 presents characteristic temperatures and deteriorating rates of 10%, 50% and 90% CO conversion rates. The T_10_s of the three CDPFs increased in different degrees. The more of the noble metal loadings, the lower of the deteriorating rate. But from No.2 to No.3 CDPFs, the deteriorating rates decreased from 0.67 to 0.63 with little drop. The T_50_s of CO for No.1, No.2 and No.3 CDPFs in fresh condition were 110.26, 118.42 and 142.83 °C and they increased to 206.60, 175.65 and 205.55 °C after 60,000 km’ aging with the deteriorating rates of 0.87, 0.48 and 0.44. The latter two had quite the same deteriorating rates, significantly lower than that of No.1 CDPF. For T_90S_, they increased little after aging of the CDPFs. The deteriorating rates had a similar changing trend with the noble metal loadings as T_50_.

**Figure 7:**
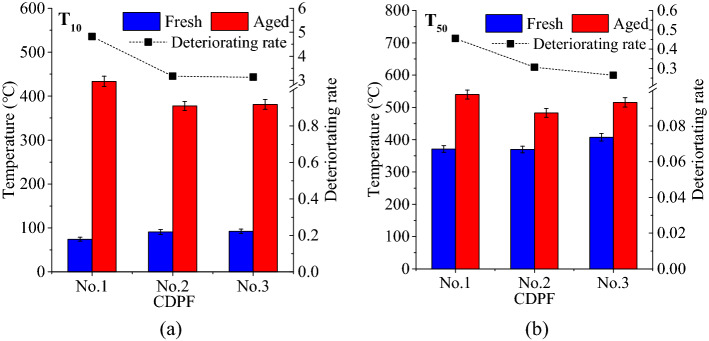
Characteristic temperatures and deteriorating rates of 10% and 50% C_3_H_8_ conversion rates (**a**) 10% C_3_H_8_ conversion rate − T_10_, (**b**) 50% C_3_H_8_ conversion rate − T_50_.

Figure 7 presents the Characteristic temperatures and deteriorating rates of 10% and 50% C_3_H_8_ conversion rates. It can be seen that the T_10_s of C_3_H_8_ for the three CDPFs were all below 100 °C, but they increased to 433.57, 377.74 and 381.24 °C for No.1, No.2 and No.3 CDPFs after aging with corresponding deteriorating rates of 4.82, 3.16 and 3.13. In terms of T_50_, the aging of CDPFs led to deteriorating rates of 0.45, 0.31 and 0.26 for No.1, No.2 and No.3 CDPFs. In general, the deteriorating rates decreased with the noble metal loadings of the CDPF, but the decline showed a decreasing trend.

**Figure 8:**
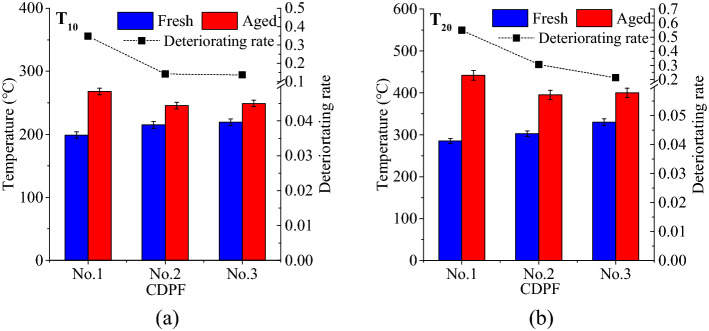
Characteristic temperatures and deteriorating rates of 10% and 20% NO_2_ production rates (**a**) 10% NO_2_ production rate − T_10_, (**b**) 20% NO_2_ production rate − T_20_.

Figure 8 shows the characteristic temperatures and deteriorating rates of 10% and 20% NO_2_ production rates. The T_10_s of the three CDPFs in fresh condition were about 200 °C and they increased to 268.03, 245.53 and 249.16 °C, respectively after aging, with the deteriorating rates of 0.35, 0.14 and 0.14. For T_20_ characteristics, the 60,000 km’ on-vehicle aging caused deteriorating rates of 0.55, 0.31 and 0.21 for No.1, No.2 and No.3 CDPFs. It can be seen that the deteriorating rates for NO_2_ production decreased with the noble metal loadings of the CDPF, but the decline also showed a decreasing trend.

### The aging performance of the different regions of CDPFs

**Figure 9:**
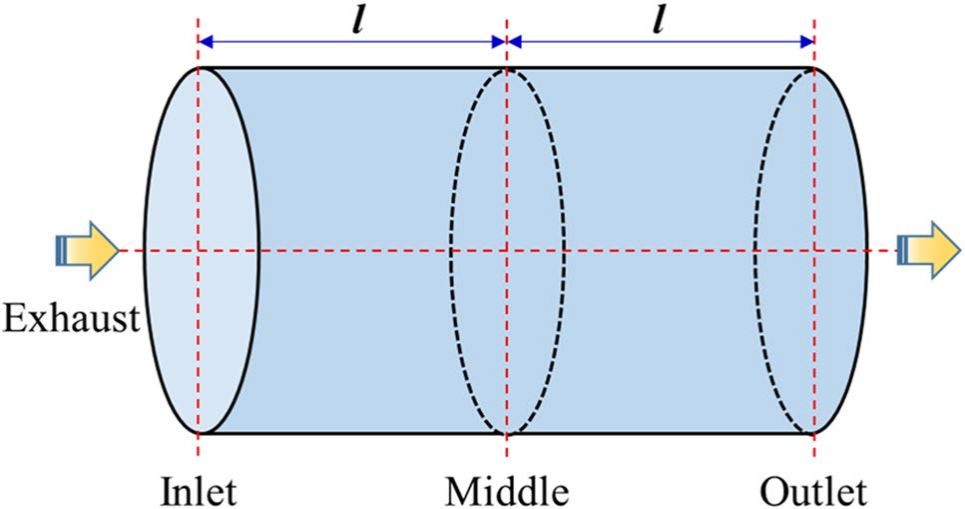
Diagram of the inlet, middle and outlet parts of the CDPF.

Due to the gradient effect of airflow and exhaust temperature in CDPFs, the aging performance of different regions of CDPFs is different. In this part, No.2 CDPF after 60,000 km’ aging was chose to study the aging performance of the inlet, middle and outlet parts as shown in Fig. 9 based on XRD, XPS and activity evaluation methods.

#### XRD and crystallinity analysis

**Figure 10:**
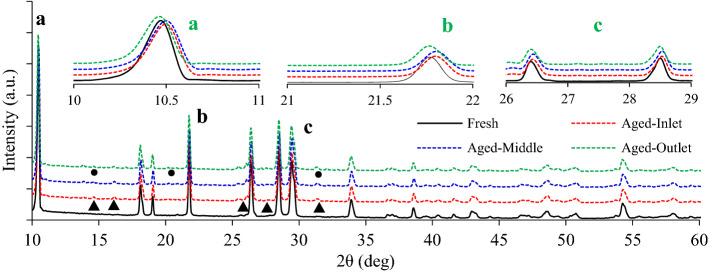
XRD results of different regions of CDPF after aging.

Figure 10 shows the XRD diffraction patterns of inlet, middle and outlet parts of the CDPF with catalyst loading of 0.71 g/L after 60,00 km’ aging. It can be seen that the XRD diffraction patterns were similar for the three parts. The characteristic diffraction peaks (marking a, b, c) all occurred in the ranges of 10°–11°, 17°–22° and 25°–31°. From the local enlargements, it can be seen that the characteristic diffraction peaks shifted towards larger angles after aging, and the offsets for the inlet and middle parts were larger, while the outlet was not remarkable.

**Figure 11:**
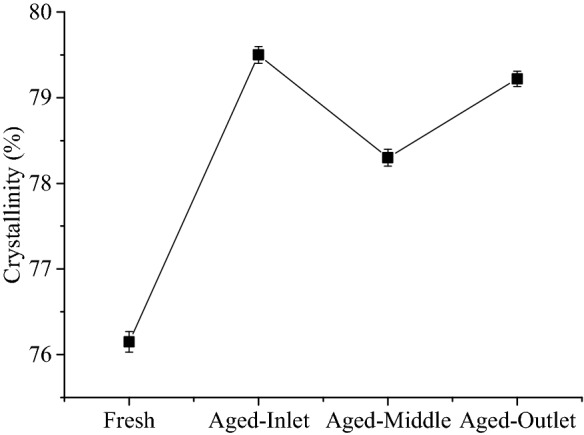
Crystallinity of the inlet, middle and outlet regions of CDPF after aging.

Figure 11 presents the crystallinity of the inlet, middle and outlet regions of the No. 2 CDPF with catalyst loading of 0.71 g/L after 60,000 km’ aging. The crystallinity increased from 76.15% to 79.50% for the inlet region. While for the middle and outlet regions, the crystallinity increased to 78.30% and 79.22%, respectively. It can be seen that the crystallinity of the inlet region was highest, followed by the outlet region and the middle region was the smallest. The increase of crystallinity is mainly caused by the sintering of noble metals and metal oxides and the formation of agglomerates with poor crystalline phase^40^.

#### Active element and valence states analysis

**Figure 12:**
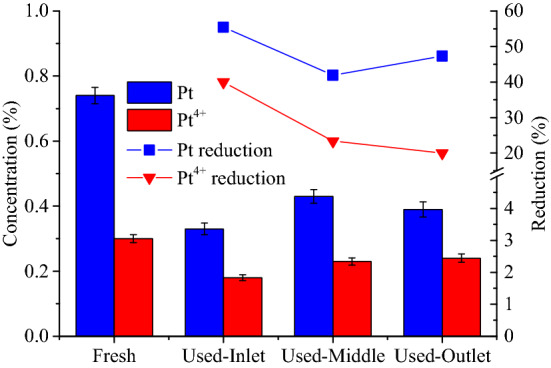
Pt and Pt^4+^ concentrations and reduction in different regions of CDPFs after aging.

Figure 12 presents the Pt and Pt^4+^ concentrations of the inlet, middle and outlet regions of the CDPF measured by XPS. It can be seen that aging led to a remarkable decrease of the Pt and Pt^4+^ concentrations. For the inlet region of the CDPF, the Pt concentration decreased from 0.74% to 0.33% with a reduction of 55.4% after aging. In terms of the Pt concentration reduction, the inlet region was highest, and the outlet and the middle region followed. It was also obtained that aging caused a 40.00% reduction of the Pt^4+^ concentration in the inlet region. While for middle and outlet regions, the reductions reached 23.30% and 20.01%, respectively. The main reason is that the CDPF aging led to agglomeration and sintering of the noble metal catalyst on the carrier, resulting in a reduction in the main active components such as Pt and Pt^4+^^41^. However, the inlet part of CDPF is always subject to higher exhaust temperature of the diesel engine compared with other parts of the CDPF, the thermal aging degree is deeper, therefore leading to larger reduction in the Pt and Pt^4+^ concentration. Although the outlet part of the CDPF bears the minimum heat load of the diesel engine, the soot trapped by the CDPF are concentrated in this part, and the oxidation of the soot will release heat, which deepens thermal aging of the CDPF outlet^42^. In comparison, the middle part of the CDPF bears the minimum heat load, the thermal aging degree is the least, causing a smaller reduction in the Pt concentration compared with those of inlet and outlet parts of the CDPF.

#### Catalytic activity evaluation

**Figure 13:**
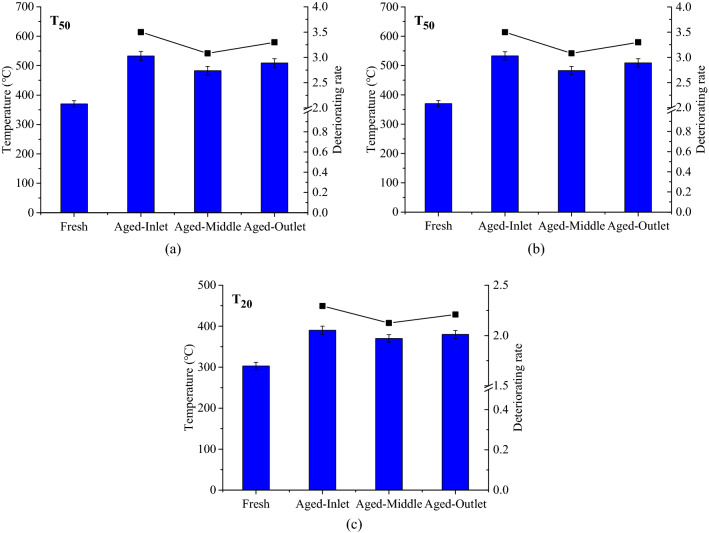
Characteristic temperatures and deteriorating rates of CO, C_3_H_8_ conversion rates and NO_2_ production rate in different regions after aging, (**a**) CO, (**b**) C_3_H_8_, (**c**) NO_2_.

Figure 13 depicts the characteristic temperatures and deteriorating rates of CO, C_3_H_8_ conversion rates and NO_2_ production rate in different regions of the CDPF after aging. It can be observed from Fig. 13a that T_50_s of CO conversion in different regions of the CDPF after aging increased and the deteriorating rates of the inlet, outlet and middle regions were 0.67, 0.62 and 0.55. In terms of T_50_s of C_3_H_8_ conversion, they increased to above 500 °C after aging for all the three regions, much higher than that in fresh condition. The deteriorating rates of the inlet, middle and outlet regions were 3.50, 3.30 and 3.08 respectively. Figure 13c shows that aging increased the T_20_s of NO_2_ production and brought about deteriorating rates of 2.29, 2.21 and 2.12 for the inlet, middle and outlet regions of CDPF.

It can be concluded that the inlet, middle and outlet parts of the CDPF shown different aging performance. The deteriorating rate of the inlet region was the highest, the following is the outlet region and the middle region was the smallest. The main reason was that the inlet region of the CDPF was nearest to the diesel engine, longtime in the higher-temperature exhaust accelerated the thermal deactivation. On the contrary, the exhaust temperature in the outlet region was relatively low, meaning less thermal deactivation^43^. However, the trapped soot in the CDPF was stacked in the outlet region due to the influence of the exhaust flow, where higher temperature would occur when the regeneration happened of the CDPF, which would accelerate the thermal deactivation of the catalyst in this region. The thermal load in the middle region of the CDPF was relatively low, resulting in less thermal deactivation compared to the other two regions.

### Microstructure and composition of the soot and ash in CDPFs with different aging mileage

In this part, the ash and soot in the No.2 CDPF was sampled and analyzed after 30,000 and 60,000 km’ using.

#### Microstructure of the soot and ash

**Figure 14:**
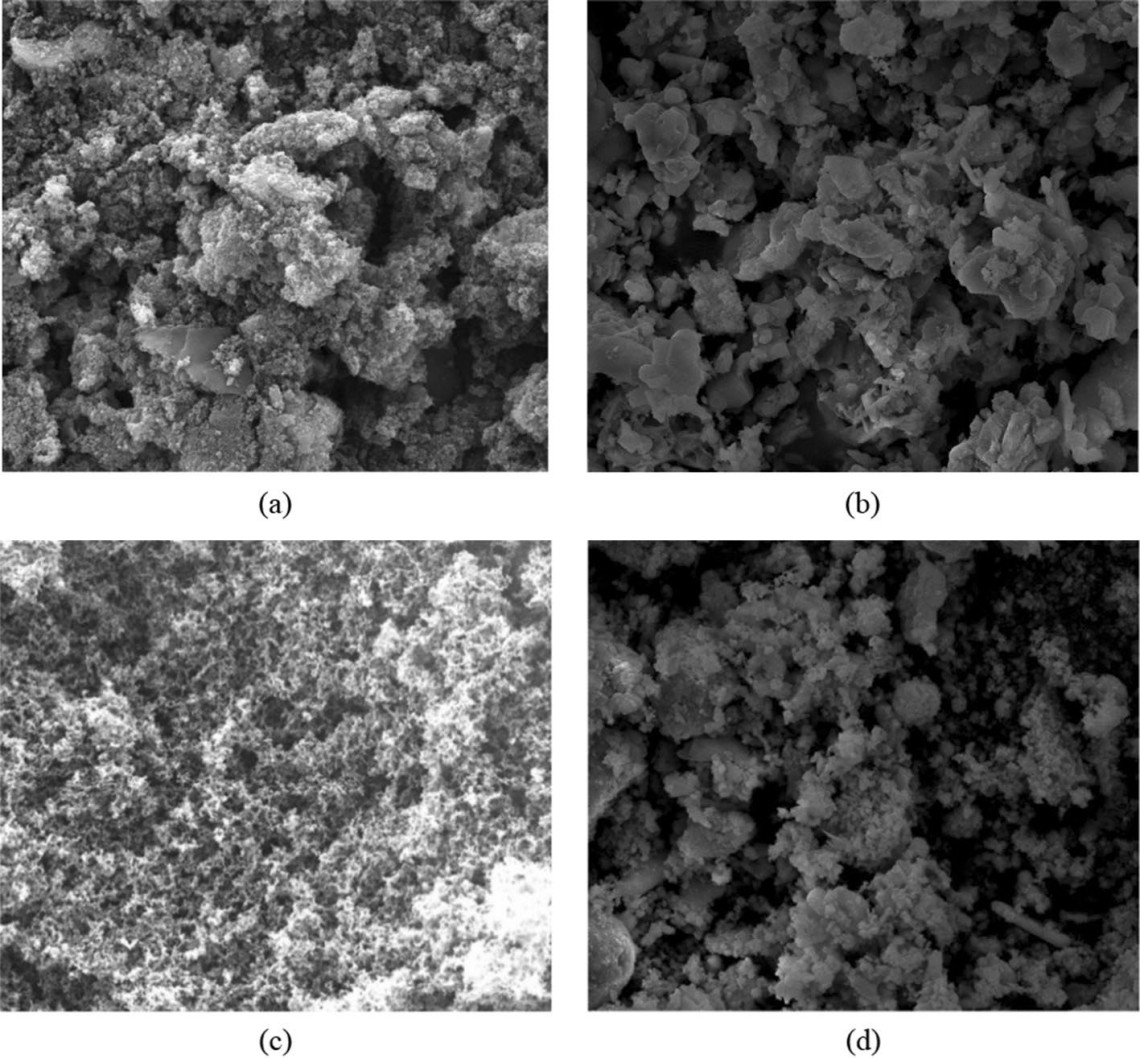
Microstructure of the soot and ash in the CDPF with different aging mileage, (**a**) soot and ash in the CDPF after 30,000 km’ aging, (**b**) ash after combustion in the CDPF after 30,000 km’ aging, (**c**) soot and ash in the CDPF after 60,000 km’ aging, (**d**) ash after combustion in the CDPF after 60,000 km’ aging.

Figure 14 shows scanning electron microscope (SEM) images of mixtures of soot and ash and the ash after the combustion of the mixture in CDPFs with different aging mileage with a magnification of 1000. It can be seen from Fig. 14a that the aggregates of the soot and ash in the CDPF after 30,000 km’ aging consisted of irregular lumps of particles. The aggregates were large and arranged loosely with obvious porous structures in between. Figure 14b shows the image of the ash of the mixture of soot and ash after combustion. It can be seen that the ash is comprised of irregular, smooth and lamellar or blocky particles, and the aggregates in the ash were small, in which the particles arranged closer. Figure 14c shows the image of the aggregates of soot and ash in the CDPF after 60,000 km’ aging. The aggregates were small with flocculent, porous surface and arranged closely each other. Figure 14d shows the image of the soot and ash mixture in the 60,000 km’ aging CDPF after combustion. The ash was clustered by irregular, globular particles and the aggregates were small and arranged more closely than that of 30,000 km’ aging.

#### Composition of the soot and ash in CDPFs with different aging mileage

**Figure 15:**
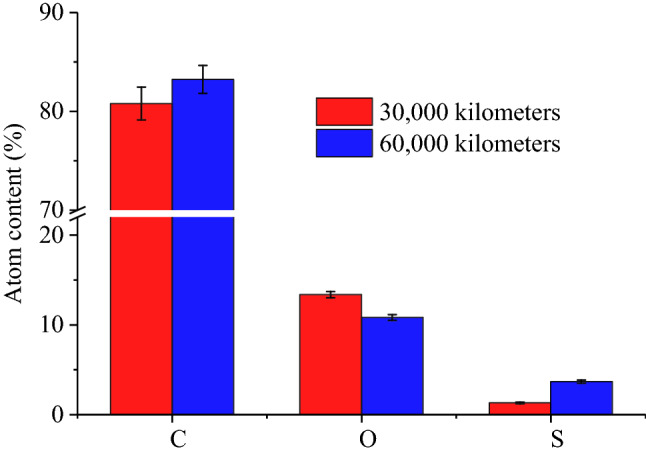
Composition and atom content of the soot and ash in CDPFs with different aging mileage.

Figure 15 presents the composition and atom content of the soot and ash in CDPFs with different aging mileage by energy dispersive spectrometer (EDS) test. The composition of the soot and ash in the CDPF mainly included C, O and S, in which C and O accounted for 80.78% and 13.35% respectively after 30,000 km’ aging. For the soot and ash in the CDPF after 60,000 km’ aging, C and O accounted for 83.24% and 10.18% respectively. It can be seen that the proportion of C increased with the aging mileage, which can be attributed to the fact that the deepening deterioration of the catalytic performance weakened the regeneration ability of the CDPF, leaving more soot unburned^30^. In terms of the S content, it reached 1.29% in the soot and ash mixture after 30,000 km’ aging of the CDPF, while after 60,000 km’ aging, the S content in the soot and ash mixture increased to 3.66%. When cleaning the ash and soot, the S content in the CDPF substrate remained 0.54%. Although the S content in the mixture of ash and soot increased about 3 times, the S content absorbed in the CDPF was very low, indicating that the CDPF has a certain tolerance to S.

## Conclusions

The effects of noble metal loadings and different regions on the aging performance of the CDPF’s catalytic property were studied using XRD, XPS and catalytic activity evaluation methods based on on-vehicle durability condition. Meanwhile, the effect of aging mileage on the microstructure and composition of the soot and ash in the CDPF was also investigated by SEM and EDS. The main conclusions are listed below:

1. The characteristic diffraction peaks of XRD patterns shifted towards larger angles and the crystallinity increased after aging of the CDPF. Besides, aging resulted in a decline of the Pt and Pt^4+^ concentrations on the CDPF surface, as well as an increase of the characteristic temperatures of CO, C_3_H_8_ conversion and NO_2_ production.
2. The noble metal loadings had significant influence on the aging performance of the CDPF. The incremental crystallinity caused by aging shown a slowdown with the increase of the noble metal loadings. And the effect of noble metal loadings on Pt and Pt^4+^ concentrations changing during aging was the same as that of crystallinity. In addition, the increase of characteristic temperatures of CO, C_3_H_8_ conversion and NO_2_ production caused by aging presented a downward trend with the noble metal loadings. The anti-aging ability of the CDPF’s catalytic performance was proportional to the noble metal loadings, but noticeably, excessive amounts of noble metals would not have the corresponding anti-aging properties. Consequently, considering the cost and performance, No.2 CDPF was the best choice of the three.
3. The aging performance of the CDPF in different regions was different. After 60,000 km’ aging, the crystallinity in the inlet region was the highest while the Pt and Pt^4+^ concentrations was the lowest compared with that in the middle and outlet regions. In addition, the characteristic temperatures of CO, C_3_H_8_ conversion and NO_2_ production were higher than the other two regions. The degree of aging in the inlet region was the deepest, followed by the outlet region and the middle region was the smallest.
4. The increase of aging mileage reduced the size of the aggregates of the soot and ash in the CDPF while increased degree of tightness between particles. The deepening of the CDPF aging could be reflected by the increase of C concentration in the soot and ash.
